# The Impact of the Risk for Sarcopenia on Frailty Among Older Adults With Physical Functional Dependency

**DOI:** 10.1093/geroni/igaa057.581

**Published:** 2020-12-16

**Authors:** Hongting Ning, Yinan Zhao, Huijing Chen, Lulu Liao, Hengyu Hu, Hui Feng

**Affiliations:** 1 Central South University, Changsha, China; 2 central south university, Changsha, China

## Abstract

Objective: 1) to assess the prevalence of the risk for sarcopenia, as well as frailty among older adults with physical functional dependency in a nationally representative sample; and 2) to identify the impact of the risk for sarcopenia on frailty in this vulnerable population in China. Methods: A total of 2,323 participants (age ≥ 60 years old) with physical functional dependency in five provinces in China were enrolled using a multistage cluster sampling scheme. Physical function was measured by the Barthel Index (BI), the risk for sarcopenia was defined as “calf circumference (< 34 cm in men, < 33 cm in women)” according to the 2019 consensus proposed by the Asian Working Group for Sarcopenia, and frailty was assessed by the FRAIL scale. Multivariate binary logistic regression was used to assess the impact of the risk for sarcopenia on frailty. Results: The prevalence of the risk for sarcopenia and frailty were 41.0% and 30.9%, respectively. Logistic regression analysis shows that the risk for sarcopenia was signiﬁcantly associated with frailty (odds ratio 1.51, 95% conﬁdence interval 1.19-1.90, p = 0.001) after adjustment for demographic and psychosocial factors, as well as health-related factors. Conclusion: This study shows that the risk of sarcopenia and frailty are prevalent, and the presence of the risk of sarcopenia increased the risk of frailty in older Chinese adults with physical functional dependency. Key Words: the risk for sarcopenia; frailty; older adults; physical functional dependency.

